# MicroRNA Expression Links Transportation Environmental Burden to Late-Stage Triple-Negative Breast Cancer

**DOI:** 10.3390/genes17070805

**Published:** 2026-07-15

**Authors:** Nubaira Rizvi, Amjila Bam, Xiao-Cheng Wu, Meng Luo, Luis Del Valle, Lang Wu, Lucio Miele, Edward Trapido, Qingzhao Yu

**Affiliations:** 1 Department of Biostatistics and Data Science, School of Public Health, LSU Health—New Orleans, New Orleans, LA 70112-2223, USA; nrizvi@lsuhsc.edu (N.R.); abam@lsuhsc.edu (A.B.); 2 Department of Epidemiology & Population Health, School of Public Health, LSU Health—New Orleans, New Orleans, LA 70112-2223, USA; xwu@lsuhsc.edu (X.-C.W.); etrapi@lsuhsc.edu (E.T.); 3 Department of Microbiology, Immunology and Parasitology, School of Medicine, LSU Health—New Orleans, New Orleans, LA 70112-2223, USA; mluo2@lsuhsc.edu; 4 Department of Pathology, School of Medicine, LSU Health—New Orleans, New Orleans, LA 70112-2223, USA; ldelva@lsuhsc.edu; 5 Department of Interdisciplinary Oncology, LSU Health—New Orleans, New Orleans, LA 70112-2223, USA; lwu3@lsuhsc.edu; 6 Department of Genetics, School of Medicine, LSU Health—New Orleans, New Orleans, LA 70112-2223, USA; lmiele@lsuhsc.edu

**Keywords:** triple-negative breast cancer, microRNA, environmental justice, transportation burden, mediation analysis, epigenetics, health disparities

## Abstract

**Background**: Triple-negative breast cancer (TNBC) is an aggressive subtype with persistent disparities in stage at diagnosis. While transportation-related exposures are increasingly recognized as environmental health risks, the biological mechanisms linking these exposures to cancer progression remain unclear. This study evaluated whether tumor microRNAs (miRNAs), which regulate gene expression and tumor behavior, mediate the association between transportation burden and TNBC stage at diagnosis. **Methods**: We analyzed 434 TNBC cases from the Louisiana Tumor Registry (2009–2019). The transportation burden from the 2022 Environmental Justice Index reflects the residential proximity to high-volume roads, railways, and airports. miRNA expression was measured via high-throughput sequencing and normalized using the trimmed mean of M-values method. Three-phase analysis (screening, individual, and multiple mediation) was performed. KEGG pathway enrichment analysis assessed downstream biological pathways. **Results**: After adjusting for age, race, body mass index, marital status, primary payer, and concentrated disadvantage index (CDI), every 0.10 (10-percentile) increase in transportation burden rank was significantly associated with a 9% increase in the odds of late-stage TNBC diagnosis (Adjusted OR = 1.09, 95% CI: 1.01–1.17, *p* = 0.021). Five miRNAs significantly mediated this relationship: downregulated hsa-let-7c-5p, hsa-let-7b-5p, hsa-miR-30a-3p, and hsa-miR-92a-3p, and upregulated hsa-miR-151a-3p. Joint mediation analysis demonstrated complete mediation (indirect effect = 0.324; *p* = 0.027). hsa-let-7c-5p was the primary independent mediator. The enriched pathways included MAPK, PI3K-Akt, Wnt, and *p53* signaling. **Conclusions**: These findings identified molecular pathways linking transportation-related environmental exposures to TNBC progression through dysregulation of miRNAs. By integrating environmental exposure assessment with tumor biology, this study advances our understanding of how the built environment contributes to cancer disparities.

## 1. Introduction

Triple negative breast cancer (TNBC) is a subtype of breast cancer based on the immunohistochemical expression of hormone receptors. It is defined by the lack of expression of three key receptors: estrogen receptor (ER), progesterone receptor (PR), and human epidermal growth factor receptor (HER2) [1,2]. From 2018 to 2022, around 13 women out of 100,000 were diagnosed with TNBC in the U.S. [3]. Among all breast cancer subtypes, it has the worst survival outcome, with a 77.1% 5-year survival rate, and the highest rates of local and regional recurrence, at 7.6% and 3.3%, respectively [1,4]. In the U.S., young women of African American and Hispanic descent are disproportionately affected, with 77% and 23% higher odds of being diagnosed with TNBC compared to their White counterparts [5]. The distinct molecular profiles, rapid progression, and limited treatment options, such as a lack of targeted therapies, make TNBC a major contributor to breast cancer–related mortality and health disparities [6].

Growing evidence indicates that environmental exposure contributes to breast cancer risk and progression, in addition to biological and social determinants. In particular, those related to air pollution and transportation infrastructure are increasingly being recognized as contributors to cancer risk and progression. The U.S. Environmental Justice Index (EJI) has defined domains of environmental exposure, including transportation proximity burden, such as proximity to airports, high-volume roads, and railways [7]. Studies examining transportation-related exposures have identified consistent associations with breast cancer risk. A Montreal-based study identified a higher breast cancer risk associated with ultrafine particle concentrations, particularly among women with ER−/PR− tumors [8]. In addition, exposure to traffic emissions during menarche and first birth doubles the risk of pre- and postmenopausal breast cancers, respectively, primarily among non-smokers [9]. Among participants with Stage I disease, residential PM exposure was associated with a 64% increase in breast cancer–specific mortality [10]. In Frankfurt, Germany, exposure to aircraft, road, and railway noise was associated with 41% increased risk of estrogen receptor-negative (ER−) tumors, particularly at 55–59 decibels (dB) [11]. A Danish cohort studies revealed higher residential exposure to road traffic noise is associated with elevated risks of ER-negative breast cancer, demonstrating up to a 28% increase in risk per 10-dB increase in noise [12]. Long Island Breast Cancer Study Project participants exposed to vehicular traffic-related PAHs were reported to have modestly increased breast cancer risk, particularly among women with ER−/PR− tumors [13]. Pooled analyses from Nordic cohorts reported that for every 10-dB increase in long-term road traffic noise, the risk of breast cancer increased by 3% [14]. These studies all suggest a strong association between both noise and air pollutants due to transportation proximity and breast cancer risk, especially for receptor-negative and aggressive subtypes.

Post-transcriptional regulators, particularly microRNAs (miRNAs), are emerging as key mediators that link environmental stress to altered tumor biology. miRNAs are small, non-coding RNAs that act as key post-transcriptional regulators of gene expression across diverse biological processes [15]. Large-scale profiling studies have identified a broad sets of miRNAs that are differentially expressed in TNBC [16,17]. Crucially, these molecules orchestrate complex signaling networks by targeting messenger RNAs (mRNAs) involved in cell-cycle control and stress responses. Dysregulation of specific miRNA families can lead to aberrant activation of canonical oncogenic pathways, including MAPK, PI3K/Akt, and Wnt, thereby driving tumor proliferation and metastasis [18,19,20,21]. Several in vitro and in vivo studies suggest that specific miRNAs contribute to the aggressive nature of TNBC [22,23].

While environmental stressors, such as transportation pollution influence cancer progression, their specific effects on miRNA expression in TNBC remain underexplored. We addressed this gap by investigating the association between proximity to high-capacity transportation infrastructure and TNBC stage at diagnosis. Using the 2022 EJI to quantify the transportation burden, we conducted multiple mediation analyses to identify specific mediating miRNAs. Finally, KEGG pathway analysis of these miRNA target genes mapped the oncogenic signaling cascades that drive disease progression.

## 2. Materials and Methods

### 2.1. Study Population

We analyzed 434 TNBC tumor samples from female patients diagnosed with TNBC in Louisiana between 2009 and 2019 [16]. Eligible participants were aged 18 years or older and had histologically confirmed TNBC. Among them, 184 (42.4%) patients were diagnosed at an early stage and 250 (57.6%) were diagnosed at a late stage.

### 2.2. Data Source

The data for this study were compiled from three distinct domains: clinical, environmental, and molecular. Clinical and demographic information were sourced from the Louisiana Tumor Registry (LTR), which is a participating registry in the National Cancer Institute’s SEER (Surveillance, Epidemiology, and End Results) program and the National Program of Cancer Registries (NPCR). The LTR routinely collects patient demographic characteristics and clinical data, including the American Joint Committee on Cancer (AJCC) stage classifications. LTR also linked its data to molecular profiles and EJI environmental metrics. The EJI provides census-tract-level variables for the cumulative impacts of environmental factors and social disadvantages on health. The indices compile information from various federal agencies, such as the U.S. Census Bureau, Environmental Protection Agency (EPA), and Centers for Disease Control and Prevention (CDC). We used variables calculated from EJI’s Environmental Burden module (EBM) [24]. All procedures and sample collections were approved by the Institutional Review Board (IRB) at LSU Health-New Orleans.

The primary outcome variable was TNBC stage at diagnosis, classified according to the AJCC on cancer staging system. Stages I and II were categorized as early-stage (early), whereas stages III and IV were categorized as late-stage disease. The early stage is set as the reference level.

The primary exposure was the transportation infrastructure domain (RPL_EBM_DOM4), derived from the 2022 EJI. This composite metric measures the transportation burden for each census tract by first calculating the proportion of the tract’s total land area that falls within a 1-mile radius of three key infrastructural features: high-volume roads (defined as continental, inter-state, or inter-metropolitan roads), railways, and airports with at least one runway [7]. These tract-level area proportions are then sorted and assigned individual percentile rankings for road proximity (EPL_ROAD), railway proximity (EPL_RAIL), and airport proximity (EPL_AIRPRT). The three percentile ranks were subsequently summed to produce a comprehensive domain score. Finally, this aggregated score is assigned a final overall national percentile rank (RPL_EBM_DOM4), ranging from 0 to 1 utilizing four significant digits, to establish the tract’s relative transportation burden [25]. For example, a census tract where 80% of its total area falls within 1 mile of both a major inter-state highway and an active railway will receive a substantially higher percentile ranking (approaching 1.0) compared to a strictly residential tract where 0% of its land falls within these 1-mile zones (approaching 0.0).

To adjust for confounders, statistical models were adjusted for the selected demographic and clinical covariates. These variables included patient age at diagnosis (in years), self-reported race (White or Black), and body mass index (BMI, kg/m^2^). Neighborhood-level socioeconomic status was captured using the Concentrated Disadvantage Index (CDI) [26]. Individual-level access to healthcare was proxied by the patient’s primary payer at diagnosis, whereas marital status was included as an established proxy for individual social support and stability.

Total RNA was extracted from 434 formalin-fixed paraffin-embedded (FFPE) triple-negative breast cancer (TNBC) tissue blocks using the Quick-RNA FFPE Miniprep Kit (Zymo Research, Irvine, CA, USA) according to the manufacturer’s protocol. Following DNase I treatment to eliminate genomic DNA contamination, RNA yield and quality were assessed using a NanoDrop spectrophotometer (Thermo Fisher Scientific, Waltham, MA, USA) and Agilent Bioanalyzer (Agilent Technologies, Santa Clara, CA, USA). Only samples with an RNA integrity number (RIN) ≥ 8 were advanced for sequencing.

Small RNA libraries were constructed using 100 ng of high-quality total RNA per sample with the QIAseq miRNA Library and QIAseq miRNA 96 Index Kits (Qiagen, Germantown, MD, USA). Library concentrations were quantified using a Qubit 4.0 Fluorometer (Thermo Fisher Scientific) prior to equimolar pooling. The pooled libraries were quantified by real-time PCR and sequenced on an Illumina NextSeq 2000 platform (Illumina, San Diego, CA, USA) utilizing 75 bp single-end reads, with a 1% PhiX Control v3 spike-in as an internal standard [27]. Raw miRNA read counts were managed using the edgeR v4 Bioconductor package.

### 2.3. Statistical Methods

**Figure 1:**
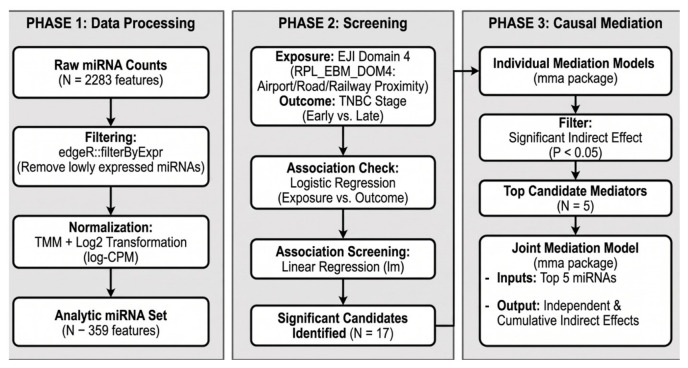
Schematic overview of the analytical framework.

To explore the relationships between the exposure variable RPL_EBM_DOM4, the 359 miRNA expression levels, and the primary outcome, cancer stage (early vs. late), analysis was conducted in three phases: (1) Data Processing, where raw miRNA counts were filtered, normalized via Trimmed Mean of M-values (TMM), and transformed into log-CPM values; (2) screening, which utilized logistic regression to validate the association between Transportation Infrastructure (RPL_EBM_DOM4) and TNBC stage, followed by linear regression to identify exposure-associated miRNAs; and (3) mediation analysis, to quantify the indirect effects of candidate miRNAs on the exposure-disease progression relationship. Figure 1 shows the analytical workflow used in this study.

In phase 1, lowly expressed features were filtered to remove non-informative transcripts and improve statistical power. This filtering step, based on experimental group sizes (early vs. late stage), reduced the dataset from an initial 2283 sequenced miRNAs to 359 high-confidence miRNAs that were retained for further analysis. To account for differences in sequencing depth and RNA composition, normalization factors were calculated using the Trimmed Mean of M-values (TMM) method. This normalization strategy corrects technical variation, assuming that most miRNAs are not differentially expressed between samples, by estimating the relative RNA production levels. Subsequently, the normalized data were transformed into log2-counts per million (log-CPM) values to stabilize the variance and satisfy the distributional assumptions required for linear modeling.

In phase 2, an unadjusted and adjusted logistic regression controlling for covariates was first conducted to confirm the statistical association between transportation exposure and clinical outcome. Subsequently, linear regression models were utilized to screen the analytic miRNA dataset, independently evaluating the association of each miRNA transcript with both exposure and outcome variables. To balance Type I and Type II errors and prevent the premature exclusion of biologically relevant pathways prior to joint mediation analysis, a nominal threshold of *p* < 0.05 was utilized for feature selection.

In phase 3, we used the general mediation analysis method which partitions the average total effect (TE) of an exposure (X) on an outcome (Y) into two components: an average direct effect (DE), an effect not mediated by miRNA, and an average indirect effect (IE), the effect mediated by miRNA [28]. The TE denotes the change in the outcome (Y) resulting from a change in the exposure (X). DE is the portion of that effect that occurs independently of the mediator, M_i_, calculated by observing the change in Y while holding M_i_ at its marginal distribution. IE is the portion of the effect transmitted through mediator M_i_. It is calculated as the difference between the total and direct effects (IE = TE − DE). All mediation analyses were performed in R (version 10.7-1), using the “mma” package. We used a multiple additive regression tree (MART) model to account for potential nonlinear associations. Confidence intervals for all effects were generated from the 2.5th and 97.5th quantiles of 2000 bootstrap samples. All statistical tests were 2-sided. A *p*-value of less than 0.05 was considered statistically significant.

In addition, to understand the broader biological effects of the five candidate miRNAs, we analyzed their associated gene pathways. First, we identified the known target genes for these miRNAs using the “multiMiR” package (version 1.30.0) in R, which searches established databases, such as miRecords, miRTarBase, and TarBase. Next, we grouped these target genes into specific biological functions using the Kyoto Encyclopedia of Genes and Genomes (KEGG) database, applying the enrichKEGG tool from the “clusterProfiler” package (version 4.16.0) [16]. To prevent false positive results from testing many pathways at once, we adjusted all *p*-values using the Benjamini–Hochberg (BH) method.

## 3. Results

### 3.1. Association Between Exposure and Cancer Stage

To evaluate the association between transportation proximity and stage at diagnosis, we first performed unadjusted and adjusted logistic regression analysis. In the unadjusted baseline model, every 0.10 (10-percentile) increase in the transportation burden rank was significantly associated with an 8% increase in the odds of a late-stage TNBC diagnosis (unadjusted OR = 1.08, 95% CI: 1.01–1.15, *p* = 0.021). This association remained similar after adjusting for age, race, body mass index, marital status, primary payer, and concentrated disadvantage index, with every 0.10 increase in transportation burden yielding a 9% increase in the odds of late-stage presentation (Adjusted OR = 1.09, 95% CI: 1.01–1.17, *p* = 0.021). Notably, none of the demographic, clinical, or socioeconomic covariates included in the adjusted model reached statistical significance (all *p* > 0.05) in this cohort. Table 1 presents descriptive statistics of the covariates.

### 3.2. Association of miRNA Expression with Exposure

**Figure 2:**
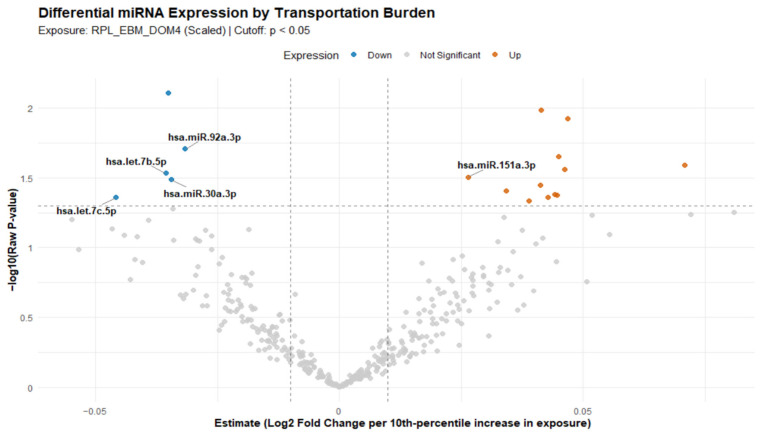
Volcano plot of the association between environmental transportation burden and miRNA expression. Estimates represent the Log2 fold-change in expression per 10-percentile increase in transportation burden (RPL_EBM_DOM4). The 17 miRNAs significantly associated with the exposure are highlighted in color, and the five specific miRNAs identified as significant mediators of TNBC stage are explicitly annotated.

An initial screening was conducted to identify potential miRNAs linking transportation burden and TNBC stage. To identify which of the 359 standardized miRNAs were associated with the exposure variable, a linear regression model was fitted for each miRNA, and the results were visualized using a volcano plot (Figure 2). This plot displays the results of all 359 statistical tests and maps the estimates against statistical significance (−log(*p*-value)). The labeled points above the horizontal dashed line represent miRNAs with a significant association (*p* < 0.05) with EJI Domain 4 transportation exposure RPL_EBM_DOM4.

A total of 17 out of 359 miRNAs were significantly associated with transportation burden, represented by the colored dots in the plot. Blue dots represent miRNAs with a negative association with exposure. That is, increase in transportation burden was associated with decreased miRNA expression. Conversely, the miRNAs with red dots showed positive association, indicating that increase in transportation burden is associated with increased miRNA expression. Their statistical associations with transportation burden using liner regression models for every 0.10 (10-percentile) increase in the transportation burden rank and with TNBC stage using logistic regression models are detailed in Supplementary Table S1.

The 17 significant miRNAs identified were selected for further analysis. We specifically highlighted the five significant miRNAs found to the significant out of the 17 in the subsequent individual mediation analysis. Among them, hsa-miR-151a-3p showed a positive association, indicating that their expression increased with higher transportation exposure. Others, such as hsa-let-7c-5p, hsa-let-7b-5p, hsa-miR-30a-3p, and hsa-miR-92a-3p, exhibited a negative association, demonstrating decreased expression in response to exposure.

**Figure 3:**
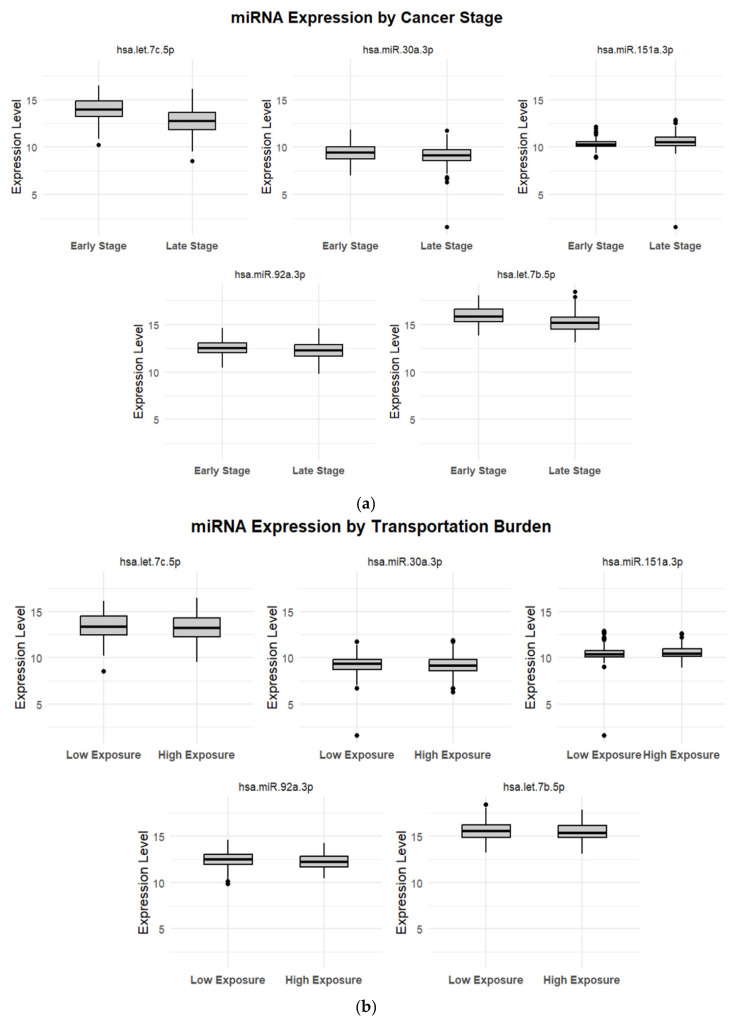
(**a**) Box Plots for the 5 significant miRNAs by TNBC Stage. (**b**) Box Plots for the 5 significant miRNAs by transportation proximity burden.

Figure 3 illustrates the differential expression of the five significant candidate miRNAs across TNBC stages and transportation burden (dichotomized at the median). The negatively associated miRNAs (hsa-let-7c-5p, hsa-let-7b-5p, hsa-miR-30a-3p, and hsa-miR-92a-3p) exhibited decreased median expression in both late-stage and high-exposure groups, with the *let-7* family displaying broader patient heterogeneity. Conversely, positively associated hsa-miR-151a-3p maintains a narrower distribution but demonstrated higher median expression in patients with a high transportation burden and in late-stage.

### 3.3. Mediation Analysis

#### 3.3.1. Individual Mediation Analysis

In phase 3, we first assessed the potential of each candidate miRNA to mediate the association between RPL_EBM_DOM4-TNBC stage. Each of the 17 exposure-associated miRNAs identified during the screening phase was included in a separate single mediator model. The indirect effect represents the portion of the effect of exposure on tumor stage transmitted through the miRNA.

In the initial individual mediation analysis, five miRNAs such as hsa-let-7c-5p, hsa-let-7b-5p, hsa-miR-30a-3p, hsa-miR-92a-3p, and hsa-miR-151a-3p exhibited statistically significant indirect effects (*p* < 0.05). Specifically, hsa-let-7b-5p showed the strongest individual indirect effect (IE = 0.354, *p* = 0.035). All significant indirect effects have the same direction as the total effect, suggesting that increased transportation burden is associated with altered expression of these miRNAs, which, in turn, increases the likelihood of late-stage TNBC diagnosis. The estimated indirect effects of the five significant candidate mediators are presented in Table 2.

#### 3.3.2. Joint Mediation Analysis

To assess the cumulative impact of these miRNA signatures, a joint mediation model was constructed that included all five significant candidates. The analysis showed a significant total effect of transportation infrastructure on the TNBC stage (TE = 0.916; *p* = 0.024), as presented in Table 3. With the five miRNAs as mediators, the joint indirect effect was statistically significant (IE = 0.324; 95% CI: 0.048–0.745; *p* = 0.027), indicating that these miRNAs collectively mediated a substantial portion of the relationship between exposure and disease severity. The direct effect of transportation infrastructure on stage became insignificant in the presence of mediators (DE = 0.591; 95% CI: −0.167–1.376; *p* = 0.147). This suggests that the impact of transportation burden on TNBC stage is primarily transmitted through miRNA alterations rather than through other unmeasured pathways. Among the individual mediators in the joint model, hsa-let-7c-5p remained independently significant (IE = 0.238; *p* = 0.031), confirming its role as a key driver within this regulatory network.

### 3.4. Pathway Analysis

We restricted our KEGG pathway enrichment analysis to the five major miRNAs that demonstrated significant mediation. Analysis of their significant target genes (Table 4) revealed that transportation-related stressors may drive TNBC progression by altering the core cellular networks. The most significantly enriched pathways governing tumor proliferation included MAPK (258 genes, adjusted *p* = 3.23 × 10^−18^), PI3K-Akt (281 genes, *p* = 2.06 × 10^−9^), and Wnt signaling (150 genes, *p* = 2.81 × 10^−11^). The miRNA signature was also strongly associated with genomic stability and cell death, including cell cycle (143 genes, *p* = 1.83 × 10^−14^), *p53* signaling (72 genes, *p* = 7.07 × 10^−11^), and apoptosis (115 genes, *p* = 1.33 × 10^−7^). Furthermore, developmental pathways linked to aggressive late-stage phenotypes such as TGF-β (92 genes, *p* = 3.86 × 10^−6^), Notch (55 genes, *p* = 1.22 × 10^−5^), and Hedgehog signaling (49 genes, *p* = 8.65 × 10^−5^) were significantly enriched. Interestingly, despite the tumors’ triple-negative status, target genes were enriched in estrogen (104 genes, *p* = 2.66 × 10^−3^) and progesterone-mediated (81 genes, *p* = 2.15 × 10^−2^) pathways, suggesting that environmental exposures may broadly disrupt systemic hormonal defenses. Collectively, exposure to higher transportation burden may advance TNBC by silencing tumor-suppressive miRNAs and activating robust oncogenic networks.

## 4. Discussion

This study provides molecular evidence linking residential proximity to transportation infrastructure with the progression of TNBC. Patients living in areas with a high transportation density (EJI Domain 4) presented with significantly advanced disease which was associated with five key miRNAs: hsa-let-7c-5p, hsa-let-7b-5p, hsa-miR-30a-3p, hsa-miR-92a-3p, and hsa-miR-151a-3p.

A central finding of this study was the identification of the *let-7* family as the primary driver of this environmental mechanism. In the joint mediation model, hsa-let-7c-5p was found to be the only significant mediator. The *let-7* family is widely recognized as a tumor-suppressive lineage that inhibits oncogenes and regulates cellular differentiation [29,30,31]. Data revealed a “double-negative” pathway: exposure to transportation proximity was associated with the downregulation of let-7c-5p and let-7b-5p, and lower levels of these transcripts were predictive of late-stage diagnosis. This resulted in a statistically positive indirect effect, suggesting that environmental stressors promote TNBC progression specifically by eroding the protective brakes normally provided by these tumor suppressors. Environmental pollutants have been shown to alter the expression profiles of tumor-suppressive miRNAs, disrupting protective cellular mechanisms against carcinogenesis [32]. The loss of these specific regulatory networks, such as the *let-7* family, promotes oncogenesis by disrupting histone ubiquitylation and increasing cancer cell migration [33].

Beyond the *let-7* family, significant mediation by hsa-miR-92a-3p and hsa-miR-30a-3p highlights how environmental exposures disrupt critical protective networks in TNBC progression. Specifically, miR-30a-3p serves as a critical tumor suppressor that normally inhibits invasion and cell cycle progression [34,35,36]. This suggests that the environmental transportation burden may promote late-stage TNBC by concurrently enhancing oncogenic signaling and silencing critical cell-cycle checkpoints. Additionally, recent studies have demonstrated that while miR-92a-3p may be elevated in patient serum, it is significantly downregulated in invasive breast cancer tissues compared to healthy controls [37]. This loss of miR-92a-3p in tumor tissue is significantly associated with higher tumor grade, advanced clinical stage, and increased cell migration [38,39,40]. Furthermore, miR-92a is essential for regulating angiogenesis and protecting normal endothelial cells against oxidative stress [41,42]. Because proximity to airports and railways chronically exacerbates oxidative stress through ultrafine particles and noise pollution, this environmental transportation burden may actively deplete these protective miRNA levels [43,44,45].

In contrast, hsa-miR-151a-3p was significantly upregulated in response to higher transportation burden. This activates stress-response pathways. miR-151a-3p is known to be highly expressed in breast tissues and has been investigated in breast cancer-related morbidities such as lymphedema [46]. The upregulation of miR-151a-3p aggressively promotes tumor cell proliferation, migration, and invasion. It achieves this by directly targeting and blocking the *p53* tumor suppressor pathway, which subsequently downregulates p21 and prevents G1-phase cell cycle arrest [47].

The biological relevance of these five miRNAs was highlighted by the enrichment of critical oncogenic and regulatory pathways. The significant involvement of MAPK and PI3K-Akt signaling suggests that environmental silencing of the *let-7* family [48]. Along with *miR-30a*, this removes critical suppressive controls on tumor proliferation, explaining the advanced disease stages in highly exposed patients. Furthermore, hyperactive MAPK signaling has been shown to actively repress *let-7* maturation via the *Myc*/LIN28 axis, driving metastatic invasion, ER-negativity, and poor clinical survival [49,50,51]. Moreover, disrupted *p53* and apoptosis pathways demonstrated that these stressors also disable cellular repair and programmed cell death [52,53,54,55]. Interestingly, despite the tumors’ triple-negative status, the enrichment of estrogen and progesterone pathways indicates that transportation pollutants may cause systemic hormonal disruptions that foster a high-risk environment for breast cancer progression.

This study had several limitations. First, although the model was adjusted for age, race, body mass index, marital status, primary payer, and concentrated disadvantage index (CDI), residual and unmeasured confounding remained possible. Environmental co-exposures correlated with transportation infrastructure such as PM2.5, were not controlled. The omission of these factors could bias the exposure, mediator, and mediator–outcome pathways, inflating the estimated mediation effects and potentially contributing to the appearance of complete mediation. Second, the exposure metric (transportation burden) is derived from the EJI at the census-tract level. It may not capture individual patterns (e.g., commuting, occupational exposures, etc.) and is subject to ecological misclassification. In addition, because longitudinal residential histories were unavailable, we could not account for residential mobility prior to the diagnosis. It is possible that some patients relocated between low- and high-burden areas during the latency period of tumor development. Third, miRNA profiling was performed on archival FFPE tumor tissue at diagnosis; pre-analytical variation, intratumoral heterogeneity, and batch effects may introduce measurement error, and cross-sectional sampling limits inference about temporal ordering among environmental exposure, miRNA dysregulation, and tumor progression. Fourth, while we identified a panel of mediating miRNAs, multiple testing and model selection could influence single-miRNA findings, despite the joint-mediation approach. Fifth, mediation analysis relies on assumptions (e.g., sequential ignorability, no unmeasured mediator–outcome confounding not affected by exposure) that cannot be fully verified; violations could bias both direct- and indirect-effect estimates. Sixth, the analysis was conducted within a single state cancer registry, which may limit the generalizability to regions with different environmental, demographic, and healthcare contexts. Finally, our use of bulk FFPE tissue sequencing rather than laser capture microdissection means our miRNA profiles reflect the entire heterogeneous tumor microenvironment rather than isolated tumor cells.

Future longitudinal studies are needed to validate the *let-7c* pathway in independent cohorts and explore the specific biological pollutants responsible for this miRNA suppression.

## 5. Conclusions

In conclusion, this study demonstrated that residential proximity to dense transportation infrastructure is significantly associated with late-stage TNBC diagnosis, mediated by miRNAs expression. The environmental downregulation of key regulatory transcripts, particularly the *let-7* family, alongside the disruption of core signaling cascades, such as MAPK and PI3K-Akt, highlights the transcriptomic response to external stressors. This research underscores the importance of integrating environmental exposure data into cancer risk assessments. Future studies are warranted to further validate these regulatory networks and explore how targeted public health interventions aimed at mitigating transportation-related environmental burdens could improve disease outcomes in highly exposed communities.

## Figures and Tables

**Table 1:** Descriptive statistics for covariates by cancer stage.

Characteristic	Overall	Early Stage	Late Stage	*p*-Value ^2^
	*N* = 434 ^1^	*N* = 184 ^1^	*N* = 250 ^1^	
Transportation Burden (EJI Rank)	0.56 (0.29)	0.52 (0.29)	0.59 (0.30)	0.02
Age (years)	62 (13)	62 (11)	62 (15)	>0.9
Race				0.11
White	226 (52%)	104 (57%)	122 (49%)	
Black	208 (48%)	80 (43%)	128 (51%)	
Body Mass Index	32 (9)	31 (8)	33 (9)	0.12
Marital Status				0.40
Single/Widowed/Divorced	215 (50%)	93 (51%)	122 (49%)	
Married	198 (46%)	85 (46%)	113 (45%)	
Unknown	21 (4.8%)	6 (3.3%)	15 (6.0%)	
Primary Payer				0.20
Private	177 (41%)	84 (46%)	93 (37%)	
Public	250 (58%)	98 (53%)	152 (61%)	
Unknown	7 (1.6%)	2 (1.1%)	5 (2.0%)	
Neighborhood Concentrated Disadvantage index (CDI)	0.03 (0.97)	−0.03 (0.99)	0.08 (0.96)	0.11

^1^ Mean (SD); n (%) ^2^ Wilcoxon rank sum test; Pearson’s Chi-squared test; Fisher’s exact test.

**Table 2:** Estimates of significant miRNAs from individual mediation analysis.

miRNA	Indirect Effect Estimate	95% CI Lower	95% CI Upper	*p*-Value
hsa-let-7b-5p	0.354	0.026	0.614	0.035
hsa-let-7c-5p	0.248	0.002	0.678	0.049
hsa-miR-151a-3p	0.148	0.016	0.378	0.025
hsa-miR-30a-3p	0.141	0.008	0.267	0.035
hsa-miR-92a-3p	0.104	0.006	0.264	0.035

**Table 3:** Estimates of effects from joint mediation analysis.

Effect Type	Estimate	95% CI Lower	95% CI Upper	*p*-Value
Total Effect	0.916	0.133	1.761	0.024
Direct Effect	0.591	−0.167	1.376	0.147
Joint Indirect Effect	0.324	0.048	0.745	0.027
Indirect via hsa-let-7c-5p	0.238	0.022	0.652	0.031

**Table 4:** KEGG pathway enrichment of 5 significant miRNAs.

KEGG Pathway ID	Description Pathway	*p*-Adjust *	Gene Counts
hsa04010	MAPK signaling pathway	3.233 × 10^−18^	258
hsa04110	Cell cycle	1.828 × 10^−14^	143
hsa04310	Wnt signaling pathway	2.811 × 10^−11^	150
hsa04115	*p53* signaling pathway	7.068 × 10^−11^	72
hsa04151	PI3K-Akt signaling pathway	2.060 × 10^−9^	281
hsa04210	Apoptosis	1.332 × 10^−7^	115
hsa05224	Breast cancer	4.120 × 10^−7^	122
hsa04350	TGF-beta signaling pathway	3.862 × 10^−6^	92
hsa04330	Notch signaling pathway	1.218 × 10^−5^	55
hsa04340	Hedgehog signaling pathway	8.645 × 10^−5^	49
hsa04915	Estrogen signaling pathway	2.656 × 10^−3^	104
hsa04914	Progesterone-mediated oocyte maturation	2.148 × 10^−2^	81

* represents the *p*-value obtained after adjustment using the Benjamini–Hochberg (BH) method, which controls the false discovery rate (FDR) to account for multiple testing and reduce the likelihood of false positives.

## Data Availability

The data that support the findings of this study are accessible upon request from the Louisiana Tumor Registry (LTR). For inquiries regarding data access and LTR data release policies, researchers are encouraged to contact Lauren S. Maniscalco at lspiza@lsuhsc.edu.

## Supplementary Materials

The following supporting information can be downloaded at: https://www.mdpi.com/article/10.3390/genes17070805/s1, Table S1: Association of 17 selected miRNAs with transportation burden and TNBC stage.
