# Supplementary material for: MicroRNA Expression Links Transportation Environmental Burden to Late-Stage Triple-Negative Breast Cancer
Source: Genes (Basel). 2026 Jul 15;17(7):805. doi: 10.3390/genes17070805 (PMC13409427; doi:10.3390/genes17070805)
Supplement: Supplementary file 1 [file genes-17-00805-s001.zip › genes-4393219-supplementary.pdf]

Table S1: Association of 17 selected miRNAs with transportation burden and TNBC stage.

miRNA	Transportation Burden (Linear Estimate)	*P*-value[a]	TNBC Stage (Odds Ratio)	*P*-value[b]
**has-miR-222-3p**	-0.0351	0.0078	0.9130	0.4586
**has-miR-26b-3p**	0.0414	0.0103	1.0365	0.7151
**has-miR-376b-5p**	0.0469	0.0120	1.0960	0.2823
**has-miR-92a-3p**	-0.0316	0.0197	0.6959	0.0026
**has-miR-127-5p**	0.0449	0.0222	0.8678	0.0889
**has-miR-187-3p**	0.0708	0.0258	1.1411	0.0099
**has-miR-376b-3p**	0.0463	0.0275	0.9401	0.4165
**has-let-7b-5p**	-0.0356	0.0293	0.4370	0.0000
**has-miR-151a-3p**	0.0265	0.0313	1.8360	0.0004
**has-miR-30a-3p**	-0.0344	0.0327	0.7023	0.0012
**has-miR.450a-5p**	0.0413	0.0359	1.1304	0.1325
**has-miR-148b-5p**	0.0342	0.0394	1.2427	0.0236
**has-miR-136-3p**	0.0442	0.0417	1.1868	0.0212
**has-miR-431-5p**	0.0447	0.0420	0.7026	0.0000
**has-miR-493-3p**	0.0428	0.0437	0.9636	0.6216
**has-let-7c-5p**	-0.0458	0.0437	0.4980	0.0000
**has-miR-503-5p**	0.0389	0.0463	0.9495	0.5290

[a] *P*-values derived from linear regression models evaluating the association between transportation burden and miRNA expression.

[b] *P*-values derived from logistic regression models evaluating the association between miRNA and TNBC stage at diagnosis.
